# Effect of Anesthesia on Oligodendrocyte Development in the Brain

**DOI:** 10.3389/fnsys.2022.848362

**Published:** 2022-05-18

**Authors:** Ningning Fu, Ruilou Zhu, Shuang Zeng, Ningning Li, Jiaqiang Zhang

**Affiliations:** Department of Anesthesiology and Perioperative Medicine, Henan Provincial People’s Hospital, People’s Hospital of Zhengzhou University, Zhengzhou, China

**Keywords:** oligodendrocytes, myelin, anesthesia exposure, developing brain, oligodendrocyte precursor cells

## Abstract

Oligodendrocytes (OLs) participate in the formation of myelin, promoting the propagation of action potentials, and disruption of their proliferation and differentiation leads to central nervous system (CNS) damage. As surgical techniques have advanced, there is an increasing number of children who undergo multiple procedures early in life, and recent experiments have demonstrated effects on brain development after a single or multiple anesthetics. An increasing number of clinical studies showing the effects of anesthetic drugs on the development of the nervous system may mainly reside in the connections between neurons, where myelin development will receive more research attention. In this article, we review the relationship between anesthesia exposure and the brain and OLs, provide new insights into the development of the relationship between anesthesia exposure and OLs, and provide a theoretical basis for clinical prevention of neurodevelopmental risks of general anesthesia drugs.

## Introduction

With the development of modern technology and an increase in the number of patients receiving general anesthesia each year, increasing attention has been paid to the effects of anesthesia on brain function, especially in infants and young children. Clinical studies have shown that there is no significant difference in cognitive function between short-term single-dose inhalational anesthesia and regional anesthesia in healthy children (Davidson et al., 2016; McCann et al., 2019). Multiple prolonged inhalational anesthesia sessions may affect the developing brain and may be a risk factor for learning and memory in children (Hu et al., 2017; Warner et al., 2018). Current clinical studies are not fully consistent due to various factors such as experimental design, sample size, and medical ethics. However, anesthesia can affect brain development and produce neurotoxicity, which has been confirmed by a large number of basic studies (Zhang et al., 2016; Makaryus et al., 2018; Sasaki Russell et al., 2019). Food and Drug Administration (FDA) has warned that long-term (more than 3 h) general anesthesia or multiple general anesthesias may harm the brain development of children under the age of three and pregnant women (Andropoulos and Greene, 2017). However, the molecular mechanism of this situation was still unclear. Therefore, more relevant research needs to be discussed. A growing body of research suggests that the effects of anesthesia on neural development may reside primarily in the impairment of glial cells, of which the development of oligodendrocytes (OLs) and myelin are the most popular. OLs differentiated from oligodendrocyte precursor cells (OPCs) are a highly specialized population of glial cells in the central nervous system whose primary function is to produce myelin and maintain the integrity of axons. This article reviews the relationship between anesthesia exposure and OLs in the developing brain. The aim was to provide a theoretical basis for clinical prevention of neurodevelopmental risks caused by general anesthesia.

## Oligodendrocytes Proliferated, Differentiated, Matured, and Myelinated

**FIGURE 1:**
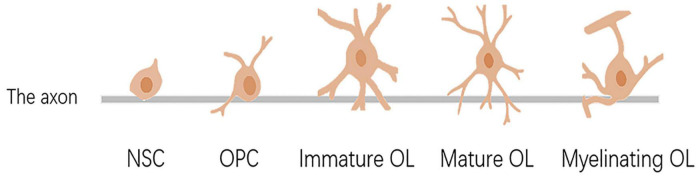
Differentiation, maturation, and myelination of oligodendrocytes. NSC, neural stem cell; OPC, oligodendrocyte precursor cell; OL, oligodendrocyte.

Glial cells are the other major cell population in the nervous tissue besides neurons, and the major glial cell types in the central nervous system (CNS) are astrocytes and OLs. OLs are produced by embryonic neural epithelial stem cells in the ventricular region of the brain, which migrate, proliferate, and differentiate into OPCs (Baumann and Pham-Dinh, 2001; Figure 1). The migration of OPCs is influenced by several factors, such as receptor-ligand binding, signaling molecules, etc., which may play pivotal roles between neural cells (Marinelli et al., 2016). Once the OPC migrated to the target site, the cell number was increased by proliferation. OPCs continue to divide until they reach a dynamic balance of cell numbers. Platelet-derived growth factor (PDGF) can promote the proliferation of OPCs, which has been reported before (Calver et al., 1998). OPCs differentiate into OLs under the action of several signaling molecules, acquire cell surface markers as cells mature, and respond to factors that regulate proliferation, differentiation, and myelination. For example, the calcium signaling pathway not only plays a role in OPC differentiation and myelination but also has a significant effect on the extension and migration of OLs in mature mice (Marinelli et al., 2016). When OPCs differentiate into OLs, they will lose their ability to divide and proliferate to become immature or mature functionalized cells. Immature OLs usually have four to five thicker processes with residual A2B5 markers on their surface. With further development, they become mature OLs and begin to express proteolipid protein (PLP) and myelin basic protein (MBP), which function to myelinate and ensheath axons (Stangel, 2002). External signals and axonal activity stimulation are closely related to any aspect of development, which is consistent with the activation of endogenous transcription factors, microRNAs, and signaling pathway proteins in OLs (Zuchero and Barres, 2015). Any abnormality in the proliferation, differentiation, maturation, and functionalization of OLs will cause neurological dysfunction, such as demyelinating disease. Of note, unlike most progenitor cells, there are still a large number of OPCs in the adult central nervous system that have the function of generating new OLs. OLs are necessary for proper neuronal development and mature neuronal function.

Yeung et al. have determined that the number of OLs in the human white matter remains at a developmental stage until 5 years of age and remains stable thereafter (van Tilborg et al., 2018). Thus, for some pediatric surgeries, exposure to anesthesia may affect OLs.

## Functions of Oligodendrocytes

Oligodendrocytes, myelinated glial cells in the central nervous system, form the myelin sheath around the nerve fiber axon and play an important role in the normal function of axon conduction. What we already know is that OLs can give nutritional support to neurons through myelin axon interaction, release various factors, and control axon growth signals. Importantly, neurons can also regulate myelination through the extracellular matrix or release various factors. For example, neurons produce neuregulin, which has pro-myelinating effects (Emery, 2010). Microglia are also important in the regulation of oligodendrocyte function and in the support of remyelination. In microglia, they play a dual role in demyelination and remyelination. The cytokine interleukin-1β(IL-1β) secreted by microglia in response to cerebral white matter injury inhibits OPC migration and myelination (Zhou et al., 2017). However, activated microglia were found to contribute to oligodendrocyte regeneration in another study (Butovsky et al., 2006). These show that microglia are dually important for the demyelination and remyelination processes in different states.

In addition, aspects such as synaptic plasticity, regulation of neurotransmitter release and metabolism, neuronal excitability, and axonal growth are also important roles for OLs (Chesnut et al., 2021). The developmental stage of the neuron, the degree of excitability, and the size of the axon can all influence myelin production (Baumann and Pham-Dinh, 2001). The myelin sheath wraps around the axon to form the myelinated nerve fibers, and the structure without a sheath between the myelinated nerve fibers is called the “node of Ranvier.” The resistance is much smaller than between the junctions. Thus, during impulse conduction, a local currency may jump from one Ranvier knot to the next adjacent Ranvier knot. This is called saltatory conduction. The conduction velocity was greatly enhanced by saltatory conduction. The myelin sheath also plays a role in the development and regulation of axon diameter and the maintenance and survival of axons. Studies have shown that OLs provide energy to axons and that, when OLs are absent, neurons are vulnerable to oxidative damage and cell death (Chamberlain et al., 2016). Finally, myelin inhibits the growth and regeneration of axons.

## Anesthesia Exposure Causes Developmental Brain Damage

The development of the human brain follows a pattern in which the brain produces important physical and chemical changes *in utero* that involve the emergence and pruning of synapses, the refinement of neural circuits, and the appearance of myelin that is not complete until adulthood. In humans, the peak of neuronal proliferation occurs at 5–25 weeks of gestation, whereas migration typically occurs at 12–20 weeks of gestation. The period from 19 weeks of gestation to 4 weeks after birth belongs to the period of rapid neuronal apoptosis, which can make the neurons appear 50–70% lower. The “peak of development” refers to the period of synapse formation starting at 20 weeks of gestation and ending at 1–2 years of age, when the number of synapses exceeds that of the adult, and the excess synapses are pruned (Tymofiyeva et al., 2014). However, myelination never stops from the second trimester until the end of life.

**FIGURE 2:**
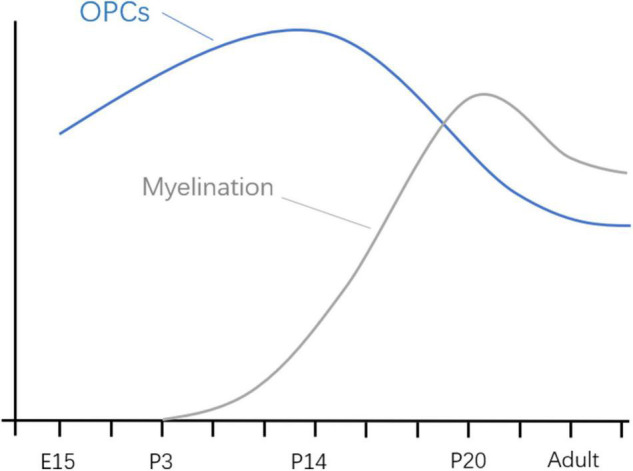
Developmental progression of OPCs and myelination in mice. E, embryonic day; P, embryonic day.

**FIGURE 3:**
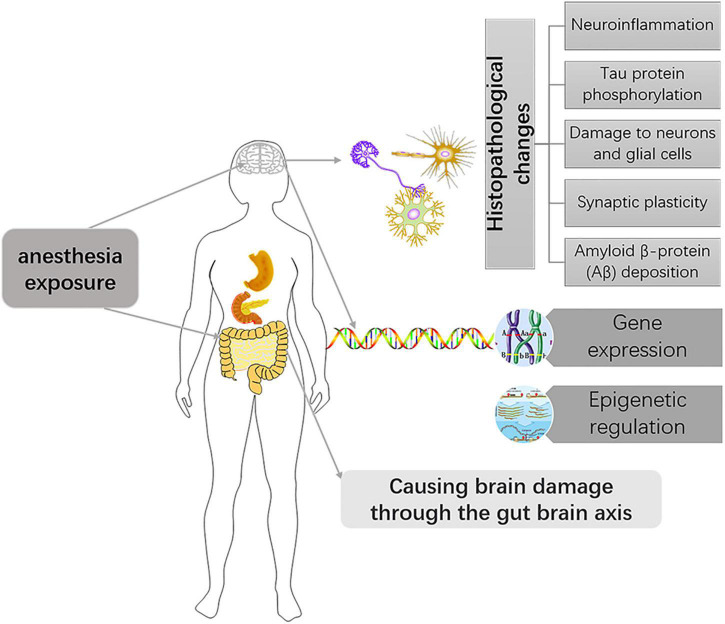
Mechanisms of anesthesia exposure induced developmental brain injury.

In different species, the time represented by the developing brain is also different. In which rodents refer to the first 2 weeks after birth, rhesus monkeys are 115 days pregnant to 60 days postpartum, while humans are 3 months pregnant to 3 years after birth (Bosnjak et al., 2016). Of course, processes such as OLs progress at different rates in different species. There is much literature on the development of OLs during human brain development. OPCs are produced beginning at 10 weeks of gestation, rapidly increasing at 15–20 weeks of gestation, and myelination of OLS starts at 30 weeks of gestation and appears mainly after birth (Jakovcevski, 2009; Bergles and Richardson, 2016; van Tilborg et al., 2018). Studies of brain development have largely been performed in rodents, making it important to understand the different time points during rodent brain development. Previous basic studies (Verity and Campagnoni, 1988; Jakovcevski, 2009) found that OPCs in the mouse brain are first detected at embryonic day 15 (E15), peak at postnatal day 14 (P14), and drop to lower levels in adults. Myelinated OLS, on the other hand, were found in the corpus callosum and internal capsule at P3 and P4, with peaks in the cortex and subcortical white matter at P20 (Figure 2). The development of neurons in the brain is preceded by the formation of a large number of neuronal precursors and determines the presence or absence of neurons depending on the amount of extracellular neurotrophic factors and whether the synaptic function is established. There have been many basic studies on the effects of anesthesia on brain development that have suggested neurotoxic effects in a time and dose-dependent manner, and the possible mechanism is as follows (Figure 3).

### Histopathological Changes

Although the mechanism between anesthesia exposure and developmental brain injury is complex and largely unknown, the key role of neuroinflammation in brain injury has been demonstrated. For example, a multicenter randomized controlled trial showed that high concentrations of interleukin-6 after skin resection during laparoscopic abdominal surgery predicted delayed postoperative neurological recovery in elderly patients (Li et al., 2021). In addition, a meta-analysis showed that the prostaglandin-endoperoxide synthase inhibitor parecoxib reduces the release of inflammatory cytokines, thereby improving early postoperative cognitive impairment (Huang et al., 2020). Another meta-analysis on dexmedetomidine also found that it can reduce perioperative inflammation and postoperative complications (Wang K. et al., 2019). These studies suggest that neuroinflammation is an important target of anesthesia exposure for damage to the developing brain.

Tau protein is a skeletal protein in the central nervous system. In the normal brain, microtubule stability relies on tau protein binding to microscopic proteins. After tau protein is phosphorylated, its binding force with microproteins is only one-tenth of that of normal tau protein. The role of maintaining microtubule stability is lost, and the phosphorylated tau is abnormally aggregated and then competes with micro proteins to bind tau protein and other related micro proteins, destroying microtubule stability, microtubule structure, and normal axon transport, causing cognitive impairment. It has been demonstrated that sevoflurane causes phosphorylation of tau in the hippocampus, which is important for the formation of abnormal cognitive function in mice (Tao et al., 2014). Another clinical study also found that when cis-phosphorylated tau was neutralized by its targeting antibody, the impaired neurological function was alleviated (Albayram et al., 2017). In animals, a relationship between the phosphorylation of tau protein and nerve injury has been demonstrated. The future still needs to further reveal the role of tau in anesthetic neurotoxicity in the developing brain.

Amyloid β-protein (Aβ) deposition can cause neurite retraction and neuronal degeneration in the mammalian brain. The pathophysiology of Alzheimer’s disease is linked to Aβ deposition, which we have previously known about (Murphy and LeVine, 2010). A basic experimental study certainly showed that sevoflurane caused neurotoxicity in the rat brain associated with intracellular deposition of Aβ(1-40) is relevant (Tian et al., 2018). The neurotoxic mechanism of general anesthesia is similar to that of Alzheimer’s disease (AD). Lu et al. (2010) also evaluated the effects of sevoflurane on young mice, and sevoflurane exposure for 6 h induced in the brain tissue of 6-day-old mice Aβ elevated levels. This suggests that sevoflurane can increase brain Aβ level even in neonatal mice. Therefore, the Aβ deposition caused by anesthesia exposure most likely is the mechanism of brain injury during development.

**FIGURE 4:**
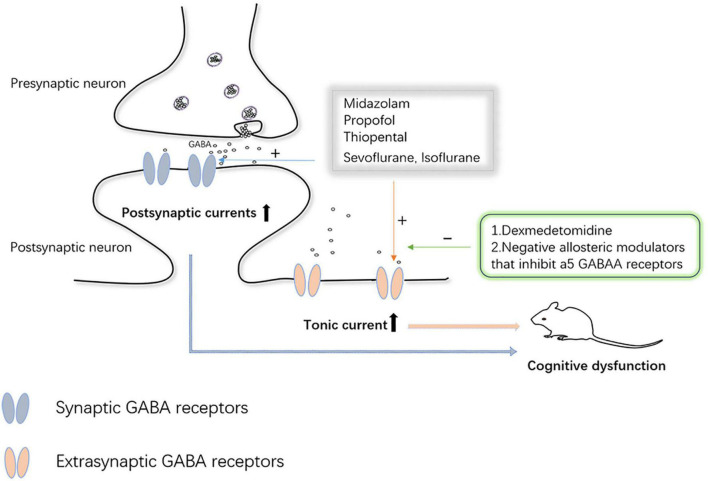
Synaptic and extrasynaptic GABA receptor-mediated inhibition of neuronal activity.

Synaptic plasticity refers to the changeable ability of synaptic connection strength and information exchange efficiency. Alterations in synaptic plasticity induced by anesthetic drugs are also one of the mechanisms underlying brain damage during the same developmental period. It has been well documented those multiple exposures can cause impairment of long-term and short-term synaptic plasticity in hippocampal neurons of the brain (Liang et al., 2017). However, it has also been documented that anesthesia with a low dose (1.2%) of sevoflurane confers improvements in learning and memory, which are similarly associated with synaptic plasticity (Chen et al., 2018). According to Zurek et al., the effects of anesthesia on synaptic plasticity in the brain are not fully understood, and changes in synaptic and extrasynaptic-aminobutyric acid type A receptor (GABA_*A*_R) activity are closely related to changes in synaptic plasticity. Even single, brief anesthetic exposures (etomidate and isoflurane) cause sustained increases in the expression and function of extrasynaptic GABA_*A*_R receptors on the cell surface of hippocampal neurons (Zurek et al., 2014; Wang et al., 2018). Bai et al. (2001) proposed that, for some anesthetics, tonic inhibition mediated by GABA_*A*_R acts in concert with synaptic inhibition mediated by other mechanisms to suppress hippocampal function during anesthesia. Several previous studies have also implicated GABA_*A*_R-mediated increases in tonic inhibitory conductance in a variety of cognitive psychiatric disorders (Down syndrome, schizophrenia, and Alzheimer’s, among others) (Caraiscos et al., 2004; Manzo et al., 2021). Caraiscos et al. (2004) declared that some anesthetics such as propofol, midazolam, and volatile anesthetics preferentially enhance tonic currents compared with synaptic currents. These all strongly suggest that GABA_*A*_R-mediated tonic inhibition plays a key role in anesthesia-induced abnormalities in cognitive function. At the same time, considering that cognitive and psychiatric disorders caused by tonic inhibition are mediated by GABA_*A*_R, negative allosteric modulators of these receptors are under intense investigation (Manzo et al., 2021). The α2 adrenergic receptor agonist, dexmedetomidine, has been extensively studied due to its neuroprotective effects. The team of Wang et al. (2018) found dexmedetomidine agitated α2 receptors and caused brain-derived neurotrophic factor (BDNF) release from astrocytes, which reduced the expression of α5 GABA_*A*_R, thereby preventing cognitive dysfunction after anesthesia (Figure 4). Nevertheless, our understanding of the role and mechanism of tonic inhibition in the nervous system is still in its infancy, but it has also attracted considerable interest from researchers.

Another mechanism by which anesthesia produces neurological damage to the brain may be the mitochondrial damage caused. An animal study has shown that exposure of neonatal rats to sevoflurane causes mitochondrial damage and neuronal apoptosis in neuronal cells (Sun et al., 2016). Anesthesia produces extensive neurodegeneration in the developing mammalian brain through the oxidative stress-related mitochondrial apoptotic pathway.

Anesthesia also has the potential to directly cause impaired development of neurons and glial cells. The effects of anesthesia on neurodevelopment have been well-documented (Creeley C. E. et al., 2013; Creeley et al., 2014; Schenning et al., 2017; Clausen et al., 2019). But the link between glia and anesthesia exposure has been little studied, and this will be highlighted later.

### Anesthesia Cause Differences in Gene Expression and Change Epigenetic Regulation

Epigenetics, defined as regulating gene expression without altering the deoxyribonucleic acid (DNA) sequence, has become an area of great interest in neuroscience. It generally refers to DNA methylation, ribonucleic acid (RNA) without any code, and histone modifications. There is a previous review detailing the relationship between epigenetic work mechanisms and anesthesia-induced neurotoxicity in the developing brain (Wu and Zhao, 2018). Volatile anesthetics have recently been shown to produce alterations in gene expression in the liver and lung (Kotani et al., 1999; Yamasaki et al., 2001). Additional studies have also found that propofol and sevoflurane anesthesia cause changes in the expression pattern of microRNAs (miRNAs) in the brain and changes in gene expression (Hamaya et al., 2000; Lu et al., 2015). A scientific study on gene expression found that neuroprotection-related genes and learning and memory-related genes in the brain undergo certain changes due to chronic or acute anesthesia exposure (Upton et al., 2020). The role of differences in gene expression and alterations in epigenetics in causing brain tissue damage during development in response to anesthesia exposure should attract our attention and thinking.

### Anesthesia Induced Changes in the Microbiota-Gut-Brain Axis

The brain-gut axis often refers to the interactions between the CNS and the gastrointestinal nervous system and is related to the immune system, the neuroendocrine system, the vagus nerve bypass system, and so on (Xu et al., 2020). The gastrointestinal tract is considered the largest organ in the immune system, and the differentiation and function of immune cells in the CNS are regulated by the gut microbiota, whose activity is essential for brain development in infants and young children. It has been shown previously that exposure to anesthesia can cause gut microbial dysbiosis (Wang L. et al., 2019; Han et al., 2021). Similarly, accumulating evidence suggests that abnormalities in the gut microflora may underlie postoperative cognitive dysfunction and postoperative mental confusion (Lin et al., 2020; Xu et al., 2020). This gives us a direction for future studies: the brain damage triggered by anesthesia exposure is not limited to the brain itself and may be related to systemic homeostasis. That is likely to be a hot area for future research in perioperative neuroprotection.

## The Relationship Between Anesthesia Exposure and Oligodendrocyte on Developing Brain

**FIGURE 5:**
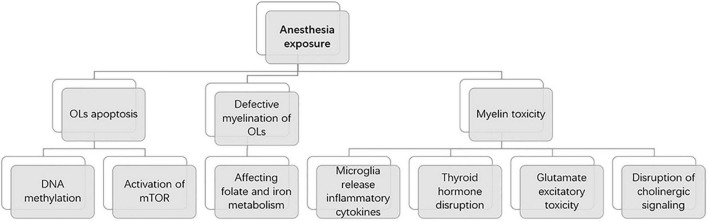
Anesthesia exposure causes oligodendrocyte injury in the developing brain.

**FIGURE 6:**
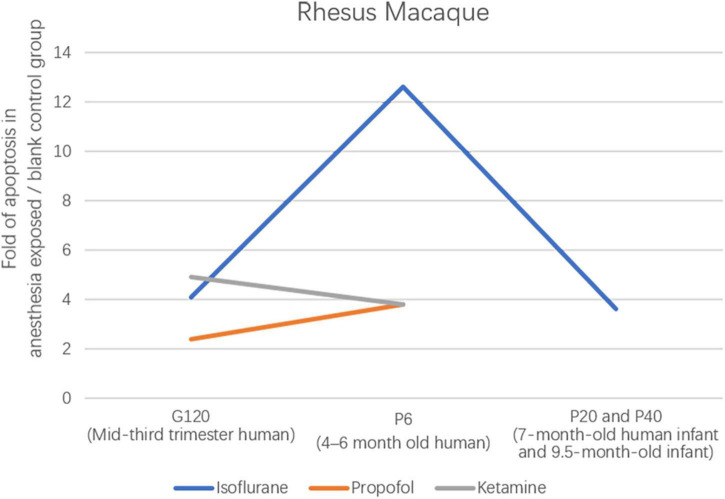
Ratio of the total number of apoptotic profiles (neurons + oligodendrocytes) in the brains of rhesus monkeys exposed to different anesthetic drugs to the total number in controls.

There are many studies on the effects of anesthesia on the neurons in the developing brain, but few studies on the relationship between the OLs and anesthesia (Figure 5). In 6-day-old (P6) rhesus macaques anesthetized by exposure to isoflurane for 5 h, OL development in the brain is massively apoptotic (Brambrink et al., 2012a). This is the first study to describe the OLs’ apoptosis induced by isoflurane, providing a basic theory for the effect of OLs on brain development under anesthesia. It is unclear how long the vulnerability window for anesthesia-induced OL apoptosis lasts, and developmental age at isoflurane exposure may affect the potential for recovery. To investigate the vulnerability of OLs to isoflurane exposure. One study found that the number of apoptotic cells of OLs was significantly reduced after *in utero* anesthesia exposure in fetal rhesus macaques at the gestational age of 120 days (G120) compared with P6 rhesus neonate anesthesia exposure (Brambrink et al., 2012a; Creeley et al., 2014). This means that in non-human primates, the neurotoxicity of isoflurane increases significantly between the third trimester and the neonatal period. As the research progresses, whether OLs in 20- and 40-day (P20 and P40) rhesus monkey brains remain vulnerable windows for 5-h isoflurane exposure has intrigued research by showing that the number of apoptotic neurons, but not apoptotic OLs, is significantly decreased compared with OLs in 6-day rhesus monkey brains (Brambrink et al., 2012a; Schenning et al., 2017). This implies that OLs remain a vulnerable window in the non-human primate brain at P20 and P40. Clinically, anesthesia for most pediatric procedures is short, so this group investigated whether 3-h isoflurane anesthesia similarly causes neuronal apoptosis in the brain, which shows similarly extensive apoptosis (Noguchi et al., 2017). From this, more experiments are still needed to examine the potential dose threshold of isoflurane and other anesthetic drugs to induce neuronal apoptosis. Similar to the results with isoflurane, 5-h propofol anesthesia exposure also induced oligodendrocyte death in G120 and P6 rhesus monkey brains (Creeley C. et al., 2013). This study showed that the vulnerable phase of OL apoptosis induced by propofol occurred before myelinated OLs. And compared to previous studies, for P6 rhesus monkeys, isoflurane was four times more neurotoxic than propofol. In P6 and G120 brains, the glial cell types selectively affected by isoflurane or propofol belong to the OL lineage (Brambrink et al., 2010). Isoflurane and propofol have been shown previously to be primary agonists of the GABA_*A*_R receptor. To further clarify the relevance of the apoptotic potential of the NMDA receptor inhibitor ketamine, Brambrink et al. (2012b) found that intravenous infusion of ketamine for 5 h in G120 and P6 rhesus monkeys similarly caused neuronal apoptosis (Figure 6). The explanation for such a strong pro-apoptotic effect of isoflurane may be that it has both γ-Aminobutyric acid_*A*_ (GABA_*A*_) agonist and *N*-methyl-D-aspartate (NMDA) receptor antagonist properties. Alcohol, like isoflurane, has both of the above characteristics. There is also evidence that alcohol induces apoptosis of OLS in the fetal brain of macaques (Creeley C. E. et al., 2013).

Besides the above-mentioned rhesus monkeys, a study in 24-h neonatal piglets (neurodevelopmentally considered comparable to human neonates) showed that 6-h isoflurane exposure induced cell death 7-fold greater than in controls (Broad et al., 2016). Isoflurane exposure for 5 h in G120 (mid-third trimester human) and P6 (4–6 months of age) rhesus monkeys resulted in 4.1- and 12.6-fold increases in cell death, implying increased isoflurane toxicity with increasing neurodevelopmental age (Brambrink et al., 2012a; Creeley et al., 2014; Broad et al., 2016).

Meanwhile, several studies on rodents have also confirmed that early postnatal anesthesia exposure in rodents affects the development of OLs (Lu et al., 2006; Kargaran et al., 2015; Li et al., 2019). Potential mechanisms involve isoflurane-induced activation of mammalian rapamycin pathway targets and an associated reduction of DNA methylation in OPCs. It is worth mentioning that it verifies the damage of anesthesia to OLs from the aspect of the proliferation and differentiation of OLs. A recent study found that early postnatal exposure to high concentrations of sevoflurane (4.9%) adversely affected the maturation and myelination of OLs in the brains of rats (Wu et al., 2020). However, the mode by which sevoflurane causes these changes remains to be further explored and validated.

In addition to the above direct effects of anesthesia on OLs causing apoptosis or developmental disorders, the latest study found that multiple anesthesia exposures caused defective myelination of OLs by affecting folate and iron metabolism, leading to impaired brain function. After maternal sevoflurane anesthesia exposure, the proliferation of cerebral OLs in the offspring was significantly reduced, myelination was inhibited, and it was finally found to be closely associated with iron deficiency in the same regions of the offspring’s brain (Zuo et al., 2020). Another study showed reduced blood folate levels in children after anesthesia, and multiple anesthesia exposures to young rats and rhesus monkeys revealed derangements in folate and DNA methylation, decreased myelin basic protein (MBP) expression, and folic acid supplementation, which promoted myelin formation (Zhang et al., 2019). It is highly likely that the study of the role of iron and folate metabolism, among others, during anesthesia will shed light on brain protection.

A recent review explains the underlying mechanisms of oligodendrocyte and myelin toxicity (Chesnut et al., 2021). These include microglia release of inflammatory cytokines, thyroid hormone disruption, glutamate excitatory toxicity, and disruption of cholinergic signaling, which ultimately lead to cell death through oxidative stress. This provides a new idea for us to study the mechanism of oligodendrocyte toxicity induced by anesthesia exposure.

Abnormal OL development induced by anesthesia exposure plays an important role in neuronal cell damage, and abnormal OL development itself can also cause neuronal damage, which is an important contributing mechanism of brain damage, suggesting that there is a potential relationship between abnormal OL development induced by anesthesia exposure and brain damage.

## Concluding Remarks and Outlook

Taken together, anesthesia is now becoming more common, alleviating patient suffering and creating favorable conditions for surgery, but multiple exposures may still have effects on the brain during development. In recent years, great progress has been made in understanding the mechanisms linking OLs to neural injury in the developing brain, which will be instructive for future basic research. It is believed that as OLs continue to be studied, they will play an important role in the prevention and treatment of anesthesia-induced damage to the brain and ultimately benefit patients receiving anesthesia.

## Author Contributions

JZ conceived and supervised the study. NF and RZ wrote the first draft of the manuscript. SZ and NL designed the figures. JZ and RZ helped supervise all aspects of the work. All authors contributed to the manuscript revision and approved the submitted version.

## Conflict of Interest

The authors declare that the research was conducted in the absence of any commercial or financial relationships that could be construed as a potential conflict of interest.

## Publisher’s Note

All claims expressed in this article are solely those of the authors and do not necessarily represent those of their affiliated organizations, or those of the publisher, the editors and the reviewers. Any product that may be evaluated in this article, or claim that may be made by its manufacturer, is not guaranteed or endorsed by the publisher.
